# Rehabilitation needs of persons discharged from an African trauma center

**Published:** 2011-11-07

**Authors:** Asare Christian, Marlís González-Fernández, Robert Samuel Mayer, Andrew J Haig

**Affiliations:** 1 Department of Physical Medicine and Rehabilitation, The Johns Hopkins University, School of Medicine, 600 North Wolfe Street, Phipps 174, Baltimore, MD 21287, USA; 2 The University of Michigan, Department of Physical Medicine and Rehabilitation, 325 East Eisenhower, Ann Arbor, MI 48108, USA

**Keywords:** Trauma, Rehabilitation, functional assessment, disability, Africa

## Abstract

**Background:**

The study prospectively assessed the functional impairments and rehabilitation needs of Africans admitted to a regional trauma center. It also acts as a pilot study to demonstrate the practical use of the Language Independent Functional Evaluation (L.I.F.E.) software in an acute hospital setting.

**Methods:**

A 5 page questionnaire was used to gather demographic data (age, sex, medical diagnosis, education, housing type, place of residency, occupation), cause of disability/injury, severity of disability or functional impairment, and rehabilitation treatment received (types of rehab, frequency of treatment, duration of therapy, follow up therapy, equipment). Functional status on discharge was evaluated with the L.I.F.E. scale.

**Results:**

84 consecutive consenting subjects were recorded. The predominant disability/injury of respondents involved the lower extremities (70%), followed by upper extremities (23%). The mechanisms of injury were largely related to auto accidents (69%). Falls made up 17% of these injuries and 14% were related to violence. Eleven subjects had disability measured using L.I.F.E and all were classified as having major disabilities. Only 14 patients (17%) received any rehabilitation therapy which consisted of only physical therapy provided at a frequency of once a day for less than one week duration.

**Conclusion:**

This study found that most persons admitted to a sophisticated trauma unit in Ghana are discharged without adequate rehabilitation services, and that the level of disability experienced by these people can be measured, even while they are still sick and in the hospital, using L.I.F.E. The implications are clear: African trauma systems must measure the long term outcomes from their treatments and provide the inpatient medical rehabilitation services that are a standard of care for trauma victims elsewhere in the world.

## Background

Around the world, the number of persons living with some form of disability is estimated at 650 million, of which 80% live in low-resource countries [1]. It has been projected that the population of disabled in low-resource countries who will require rehabilitation will reach 125 million by 2035 [2]. There are approximately 78 million people with disability in Sub-Saharan Africa [3]. This number is on the rise due to the increase in chronic disease, trauma (falls, motor vehicle accidents, injuries, violence, and wars), life sustaining advances in medicine, and due to the ageing population [1].

Perhaps the most important modifiable risk factor for this increase in disability is trauma. Most cases of trauma in low-resource countries result from automobile accidents. High income countries own 89% of the world's automobiles and yet suffer only 24% of auto accident deaths [4]. In contrast, low resource countries own 11% of the world's automobiles and yet 76% of deaths from car accidents come from these regions [4]. The WHO Guidelines for Essential Trauma recognizes that trauma frequently causes disability and is amenable to rehabilitation but the lack of physical medicine and rehabilitation physicians (physiatrists) worldwide “prevent this recommendation from being deemed essential” [5].

Rehabilitation after trauma is a serious and rising problem for low-resource countries, yet it has not been widely studied. Since disability tends to be environmentally and regionally influenced, research into this topic needs to be done at locations where resources are representative but optimal. Ghana serves as an important model for exploring issues of trauma rehabilitation, because although it is a low-resource country, it has highly sophisticated trauma care at a few university-associated hospitals. In contrast to high-level trauma care, and typical of all other African countries, medical rehabilitation resources are almost non-existent [6]. The study of rehabilitation needs among African patients whose acute trauma care is excellent helps clinicians and policymakers understand whether their strategies and allocation of resources are appropriate.

Finally, measurement of disability and rehabilitation needs is difficult in Africa because of language and literacy barriers. A recent development, the Language Independent Functional Evaluation (L.I.F.E.), may be a useful tool for scientists and clinicians to evaluate the functional needs of persons in African hospitals [7]. This computer-animated tool, designed by a team of American and African investigators, has been shown to have good face, content, and construct validity as used in the United States, Mongolia, Colombia, and Ghana [8–11]. However it has not been used in the acute hospital setting.

The current study prospectively assesses the functional impairments and rehabilitation needs of Africans admitted to a regional trauma center. It also acts as a pilot study to demonstrate the practical use of the L.I.F.E. in an acute hospital setting.

## Methods

### Sample selection

We designed a cross-sectional study of hospital inpatients. All patients age 18 and older admitted to the trauma ward who were medically stable and cognitively intact to provide informed consent participated in the interview process. These inclusion criteria maximize the range of participants in terms of demographics, injury/disability type, functional impairments, and appropriateness for rehabilitation. Patients with thoracic and abdominal trauma were excluded from the study to ensure that patients were appropriate to participate in rehabilitation.

Participation was voluntary, without compensation. Written consent was obtained based on literacy level. Patient who were illiterate gave consent with thumbprints, a substitute for signature in Ghana. Patient privacy was protected during collection and processing of data. A random number was assigned to each patient and the data was collected anonymously. Clearance for this work was obtained from the Committee on Human Research, Publications and Ethics, Kwame Nkrumah University of science and Technology (KNUST) and Komfo Anokyi Teaching Hospital (KATH), Kumasi, Ghana; Medical College of Wisconsin Institutional Review Board, Milwaukee, WI, USA; and the Johns Hopkins University Institutional Review Board, Baltimore, MD, USA.

### Setting

The study was carried out at Komfo Anokye Teaching Hospital (KATH) in Kumasi, the capital of the Ashanti region of Ghana. Kumasi has an estimated population of 650,000[12]. KATH is the only tertiary hospital in this region of the country; it is also the main teaching hospital for the School of Medical Sciences, Kwame Nkrumah University of Science and Technology (KNUST). With 450,000 out-patient visits annually and 42,000 in-patients admissions, KATH with it 1000-bed capacity is the second largest hospital in Ghana. The 4 trauma wards include general trauma wards for women and men (41 and 62 bed capacities respectively) and senior male and female trauma wards for more affluent men (30 beds) and women (20 beds). Persons with burn injuries are not admitted to these trauma wards thus were excluded from study. Typical of almost all African tertiary care centers, KATH has a physiotherapy group and basic orthotic capacity, but there is no inpatient rehabilitation ward and there are no physiatrists, occupational therapy, speech-language pathology, or other rehabilitation related professionals.

### Interview process

Data collection was conducted from April 14th 2009 to April 28th 2009. Patients were interviewed in English or in their native language, Akan, by investigator AC who is a native speaker. A 5-page questionnaire in English with Ashanti vernacular translation was administered to participants. The questionnaire covered demographic data (age, sex, medical diagnosis, education, housing type, place of residency, occupation), cause of disability/injury, severity of disability or functional impairment, and rehabilitation treatment received (type of rehab, frequency of treatment, duration of therapy, follow up therapy, equipment).

### Variables

A scale developed by Mock et al. [12] was used to classified disability type. This included: A) Self-care limitations: unable to feed or care for self. B) Mobility restrictions: Unable to walk at all, without artificial device or assistance of others. C) Major disability: Inability to grasp with a hand or inability to walk for more than 1/4 mile. D) Minor disability: Some limitation of function, but not severe enough to be classified as major. Disability was also assessed by extent to which injury/disability interferes with social life, sleep, lifting, and occupation.

Functional status on discharge was evaluated with Language Independent Functional Evaluation (L.I.F.E.) software [7]. L.I.F.E. consists of computer animated representation of the 10 functions which are assessed in the commonly used Barthel index [13]. L.I.F.E. software portrays basic functions such as bathing, feeding, toileting, grooming, dressing, and transferring on a computer screen. The touch screen allows patients to choose the level of assistance they require (partial assistance, total assistance, independent) to perform these functions. L.I.F.E. has been shown to have excellent content, face, and construct validity in population-based studies [8,9] and has been validated in Ghana as a measure of function with good reliability in community dwelling Ghanaian populations [7].

The Medicare model set by the Center for Medicare and Medicaid Services (CMS) for determining rehabilitation needs in the United States was used to determine which patients would qualify for acute inpatient rehabilitation services if available. The Medicare criteria for medical rehabilitation are based on an extensive evidence-base demonstrating the cost-effectiveness of rehabilitation interventions.

The admission criteria are based on type of injury, admission evaluation process, and the amount of therapy a person receives in acute inpatient rehabilitation facility. Major multiple trauma is one of the 13 primary diagnoses required to comprise 75% of the admissions to acute rehabilitation facilities in the US. To qualify for an admission to acute inpatient rehabilitation, patients must require and be able to benefit from at least three hours of rehabilitation therapy including physical therapy, occupational therapy or speech therapy. Patients must have a total of 15 hours of therapy over a week, usually three hours daily Monday through Friday. Recreational therapy, music therapy, respiratory therapy, neuropsychology or cognitive therapy can be used to satisfy the requirement for patients to receive intensive rehabilitation therapy [14].

## Results

### Demographics

Eighty four consecutive consenting subjects were recorded. Sixty six percent of the respondents were male. The majority (63%) of the respondents had high school education or less. More than one half of the respondents resided in rural areas (55%) and the majority (95%) lived in single story house. Most respondents were between the ages of 18-44 (67%). Seven patients were from senior wards vs. 77 from regular floors.

### Disability/injury type

The predominant disability/injury of the respondents involved the lower extremities (70%), followed by upper extremities (23%). The mechanisms of injury were largely related to auto accidents (69%). Falls made up 17% of these injuries and 14% were related to gunshot wounds or assaults.

### Severity of disability/functional limitations

Based on the Mock classification, mobility restrictions accounted for 70% of disabilities, minor disability 16%, self-care limitation 11%, and 2% had major disability.

### Therapy following injury

The rehabilitation services received by these patients are described in Table 1 as compared to what would be recommended in the United States. Out of the 84 participants, only 14(17%) received some form of rehabilitation therapy. Only physical therapy was provided, and for the majority once a day for less than a week. There was no occupational or speech therapy provided. Fifty-seven percent (4 out of 7 patients) from the senior male and female wards received therapy versus 13% (10 out of 77) from regular floors. In contrast, using the Medicare model all of these patients would have qualified for acute inpatient rehabilitation services.

**Table 1:** Comparison of Recommended vs. Received Rehabilitation Services by Trauma Unit Patients in Ghana, 2009, n=84

Rehabilitation need	Recommended	Received
Acute inpatient rehabilitation unit+	84 (100%)	0 (0%)
Physical Therapy*	84 (100%)	14 (16%)
Occupational Therapy*	84 (100%)	0 (0%)
Assistive device #	84(100%)	15(18%)

+Acute inpatient admission is defined as qualifying for admission to a United States Medicare-exempt acute medical rehabilitation inpatient unit.

*Therapy requirements are based on treatment customarily provided in an American hospital.

#Assistive devices at discharge are considered required if they would likely improve some L.I.F.E. function by 1 point for more than 1 month.

Of the 84 respondents, only fifteen received some assistive device for temporary use during hospitalization. These consist of five crutches, three wheelchairs, two orthosis (knee brace), one walker and four straight canes. These assistive devices were shared between patients. For example, patients needing wheelchair transport to the bathroom had to wait until one was available. All those who received the equipment said it was helpful. Only 8% of the respondents said a device was prescribed for them upon discharge from the hospital. Fifty percent of patients who received therapy were given follow up therapy upon discharge. More than 90% of patients had some support system to take care of them at home upon discharge. Unfortunately 92% of caregivers received no training in the hospital and have no training in care for the patients, patient prognosis, or injury complications.

### Functional status on discharge (L.I.F.E)

**Figure 1:**
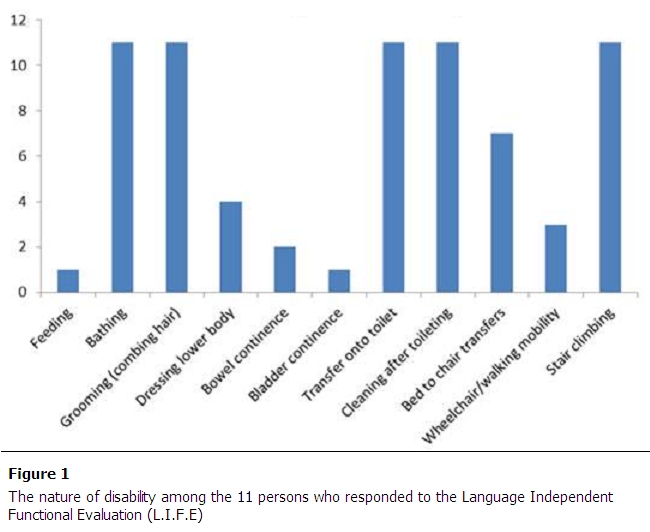
The nature of disability among the 11 persons who responded to the Language Independent Functional Evaluation (L.I.F.E)

We measured disability at discharge using L.I.F.E. on 11 subjects (13%). All patients evaluated with L.I.F.E. had significant disabilities as compared to 18% identified as having minor disability by the Mock scale (Table 2). Among these persons, 9 of 11 had purely orthopedic impairments. Yet, as Figure 1 and Table 2 points out, all had impairments in bathing, grooming, stair climbing, toilet transfers and hygiene after toileting, with a substantial number disabled on other life activities (Table 3).

**Table 2:** Comparison of Mock Trauma Disability Scale and Language Independent Functional Evaluation (L.I.F.E). Findings in a Sample of 11 Subjects, 2009, n=11

Subject/Diagnosis	Mock Scale	L.I.F.E Score	L.I.F.E disabilities
1.Left tibia and Fibula fx	Mobility restriction	16	Below* plus bladder incontinence
2.Brain Injury, leg fx	Mobility restriction	11	Below* plus dressing lower body, bed to chair transfer
3.Left Femur fx	Mobility restriction	14	Below* plus bed to chair transfer
4. C5-6-7 fx	Minor disability	0	Below#
5. Right Femur fx	Mobility restriction	15	Below*, plus bed to chair transfer, walking mobility
6.Left foot toe lost	Mobility restriction	17	Below*
7.Lt upper arm fx	Mobility restriction	16	Below* minus stair climbing
8.Right tibia, Fibula, and femur fx	Mobility restriction	7	Below* plus dressing lower body, walking mobility, bed to chair transfer
9.Lt Femur fx	Minor disability	17	Below*
10.Lt Femur fx	Mobility restriction	16	Below*
11.Pelvic Fx	Mobility restriction	16	Below* plus bed to chair transfer

*All of these subjects had impairments in bathing, grooming, stair climbing, transfers onto toilet and cleaning after toileting.

# Impairments in all 11 domains of function. Fx: Fracture

**Table 3:** Functional status on discharge of 11 subjects measured by the Language Independent Functional Evaluation (L.I.F.E) (Details)

Functional limitations	L.I.F.E Response by Subject											% IN
	
	1	2	3	4	5	6	7	8	9	10	11	
Feeding	I	I	I	D	I	I	A	I	I	I	I	81
Bathing	A	A	A	D	A	A	D	D	A	A	A	0
Grooming	A	A	A	D	A	A	D	A	A	A	A	0
Dressing lower body	I	A	A	D	I	I	I	D	I	I	I	64
Bowel Continence	I	D	I	D	I	**I**	I	I	I	I	I	81
Bladder Continence	I	I	I	D	I	I	I	I	I	I	I	91
Transfers onto toilet	A	D	D	D	A	A	A	D	A	D	A	0
Cleaning after toilet	A	A	A	D	A	A	A	D	A	A	A	0
Bed to chair transfer	A	D	A	D	A	I	I	D	I	I	A	36
Wheel chair/walking mobility	I	I	I	D	A	I	I	D	I	I	I	73
Stair climbing	A	A	A	D	A	A	I	D	A	A	A	0
Total L.I.F.E Score	16	11	14	0	15	17	16	7	17	16	17	

D= Dependent (score=0) A: Partial assistance (score=1) I: Independent (score=2), IN=Independent

Subjectively, the first author, an American-trained native Ghanaian physiatrist, found that most of the patients in the study had no idea of what rehabilitation medicine meant. The lack of knowledge about rehabilitation medicine was not related to age, sex, rural vs. urban, or literacy level. It became clear from the beginning that physical therapy, occupational therapy and speech therapy were unknown to these populations even when a translation like “someone who comes and stretch you” was used. This was how physical therapy was described and perceived in the vernacular, however the very concept that injured limbs benefit from rehabilitation was not understood among these trauma victims.

## Discussion

This is the first study to objectively review the rehabilitation needs in a tertiary care trauma center in Africa. The results showed that the great majority of people admitted to the trauma service in Ghana did not receive any rehabilitation services, and the few that did received inadequate services. We also demonstrated that L.I.F.E. can be a viable option to measure function among African hospital patients.

We were struck by the fact that all patients recruited in the study from the trauma units would have met criteria for acute inpatient rehabilitation admission in the United States. Even patients with non-weight bearing status might have had an early rehabilitation phase involving maintaining joint ROM (range of motion), muscle strength, sitting balance among others. After the medical diagnoses and functional impairments were categorized, a model for determining rehabilitation needs was applied. This model demonstrated that Medicare (the US government's insurance program for people above 65 years of age) would approve and finance rehabilitation care for all the cases studied [14]. Medicare criteria for inpatient rehabilitation are in fact more stringent than almost every European or industrialized Asian country.

There was also a lack of appropriate assistive devices in the hospital for patient use and they were rarely prescribed upon discharge. The need for assistive devices is based on the investigators’ proposal that issuance of a specific assistive device (wheelchair, crutch, brace, etc.) would immediately improve function by one point in one of the basic functional abilities outlined in the L.I.F.E., and would be necessary for more than 1 month.

### Trauma Rehabilitation

To our knowledge, the only review of rehabilitation in Ghana was recently published by Tinney et al. [6]. They have proposed a model for sustainable medical rehabilitation which can serve as a platform for evaluating rehabilitation systems in other countries. The majority of work done on rehabilitation in Ghana and low resource countries has focused on Community Based Rehabilitation (CBR) in rural areas [1–3,17–20]. In general, little research exists on the need for rehabilitation medicine in low resource countries [1,21,22]. Perhaps the most compelling statistic is that published in a special communication of the International Rehabilitation Forum (IRF) published simultaneously in 5 medical journals around the world [23]. This communication reported that every major continent has 10,000 or more physiatrists except for sub-Saharan Africa where only 6 physiatrists were identified to serve the 750 million people living in that part of the continent.

There is research on the subject of injury-related disability and orthopedic care in Ghana but few authors explore rehabilitation care. Ghana has more than 1 million people with disabilities [3], of whom only 5% receives rehabilitation services [6]. The majority of trauma-related disabilities involve lower extremity impairments which can be prevented by improving orthopedic and rehabilitation care [15,16].

The absence of medical rehabilitation at KATH is likely the result of lack of knowledge. In Ghana the Essential Trauma Care projects have been important in improving trauma care [27]. KATH, for example, recently opened a greatly expanded state of the art trauma center. Yet at KATH there were no provisions to expand the already inadequate rehabilitation services. As a result it is likely that trauma center length of stay at KATH is longer than it should be, and, as shown here, many trauma patients are discharged with untreated disabilities.

### Use of the L.I.F.E in a hospital setting

The self-report of disability by the person receiving acute trauma care is of note. Most research into the needs of people with disability focuses on people living in the community [6,12,24]. This representation of medically stable people alone creates a bias against those who would benefit from acute rehabilitation services. Furthermore, people who are medically stable are subject to recall bias regarding prior rehabilitation needs [25]. We chose to study acute trauma patients to reduce recall bias and to provide a more accurate account of the needs of those who will directly benefit from our research. The ability to eliminate literacy and language translations in functional assessment with L.I.F.E. was appropriate and practical in this population considering the fact that majority (63%) of study subjects have less than High School or no formal education at all, consistent with the educational levels in Ghana [26].

The Mock scale was used to classify all patients admitted, and L.I.F.E was used on 11 patients who were being discharge home during the time of the study. The current experience shows the viability of the L.I.F.E. in the environment of a trauma center where English is not always spoken and the literacy level is low. While global disability scales such as that proposed by Mock have an advantage in terms of simplicity, it is obvious in our research-that they are not detailed enough to show clinicians and policymakers the functional impact of injuries or the potential requirements for medical rehabilitation interventions. Though only 2% and 16% had classification of major and minor disability respectively, based on the Mock scale, there were substantial functional impairments and limitation in the group, as outlined by L.I.F.E. The Mock scale fails to identify impairments that can significantly impact a person's ability to function in society, carry on their occupation, social life, and ADLs.

### Implications for African trauma services

Our results demonstrate that, most people on these wards had significant trauma, leading predictably to substantial disability. However few of them received any rehabilitation services at the level considered appropriate in other systems of care, and there was no possibility of providing the multidisciplinary rehabilitation inpatient services that are standard of care for trauma systems elsewhere, since there are no professionals with the appropriate expertise.

The core problem is that trauma policy is biased by the use of short term success metrics such as mortality and hospital length of stay. In contrast the real cost of trauma should be calculated from a lifetime of significant untreated disability. Trauma centers that do not have comprehensive rehabilitation have been shown to have worst outcomes [28–31]. Failing to prevent permanent disability in those acutely stabilized after trauma has not only economic, societal, and clinical implications but moral and ethical implications. In most of Africa those who allocate resources to trauma care do not measure the cost of long-term disability and the loss of productive life-years, thus resources are not allocated to prevent it. It is critical that those who fund and design African trauma systems focus on the long-term cost of trauma and trauma-related disability to optimize outcomes through medical rehabilitation.

An overwhelming literature supports multidisciplinary, physiatrist-driven inpatient hospital rehabilitation programs as the standard of care for significant trauma ranging from amputation to spinal cord injury. In a study of 124,421 persons admitted to trauma centers across Washington State, USA over a 13 year period, Davidson et al. evaluated the risk of death after hospitalization. The hazards ratio (compared to those who went home without need for any assistance) was 2.88 for those who went to physiatrist-directed rehabilitation, 3.78 for those who went home with assistance, but not inpatient rehabilitation, and 8.92 for those who were admitted to sub-acute facilities without comprehensive rehabilitation [31].

As proposed by Kosar, et al (2010), an integrated multi-trauma rehabilitation service approach is more cost-effective than conventional multi-trauma care service. Their integrated model encompasses early rehabilitation including: 1) early involvement of the physiatrist in the hospital; 2) shorter stay in hospital with early transfer of multi-trauma patients to specialized trauma rehabilitation units; 3) injury-specific physical and psychosocial treatment; 4) individual goal settings 5) psychological and social counseling; and 6) integrated and coordinated treatments between trauma surgeons and rehabilitation physicians [30].

The lack of post-trauma rehabilitation in Africa can be directly tied to the absence of physiatrists on the continent. Since most African physicians and policymakers have never met a physiatrist, they often presume that rehabilitation is equal to physiotherapy. Yet most components of medical rehabilitation fall outside of the expertise of physical therapists. African therapists (who also typically have never worked in a medical rehabilitation setting) are at a distinct disadvantage when advocating for interventions ranging from spasticity injections to antidepressants to swallowing evaluations. In the short term therapists and trauma surgeons can patch the gap, but in the long term they need to advocate for institutions of higher education in Africa to train the physicians and other specialists who can bring rehabilitation services up to standard.

### Study Limitations

These findings need to be interpreted in light of the methodology used. Only a small number of subject participated in L.I.F.E data collection. The study was done at one medical center, with short duration of data collection, limiting generalizability. However, findings at KATH can be extrapolated to other parts of Ghana, since the demographics, causes of injury, and availability of rehabilitation services are not likely to be different. In fact, many parts of Africa have fewer rehabilitation resources and less sophisticated trauma care. Thus these findings likely represent a best-case scenario.

KATH being the only tertiary hospital in the upper half of Ghana, recruited participants from a wide population group. Members from most major tribes in Ghana (Ashanti, Fanti, Ga, Ewe, and Hausa) were represented in our study population. This by no means is suggestive that the study evaluated all the tribes or languages in Ghana but the above represents languages spoken from all corners of the country. Patient from different wards, i.e. general trauma floor and senior wards represented a wider socioeconomic range. Prospective studies of trauma patients from admission to discharge can help us better characterize trauma-care cost as compared to costs in optimized trauma-care systems with early rehabilitation while taking into account the cost of lost productivity.

## Conclusion

This study found that most persons admitted to a sophisticated trauma unit in Ghana are discharged after receiving little or no rehabilitation services, and that L.I.F.E can be a useful tool to measure the level of disability experienced by trauma patients even while they are still sick and in the hospital. The implications are clear: African trauma systems must measure long-term treatment outcomes and provide inpatient medical rehabilitation services that are standard of care for trauma victims elsewhere in the world.
